# Synthesis of iron-doped TiO_2_ for degradation of reactive Orange16

**DOI:** 10.1186/2052-336X-12-19

**Published:** 2014-01-09

**Authors:** Mojtaba Safari, Rasoul Talebi, Mohammad Hossein Rostami, Manouchehr Nikazar, Mitra Dadvar

**Affiliations:** 1 Department of Chemical Engineering, Amirkabir University of Technology, Tehran, Iran

**Keywords:** Iron-Doped TiO_2_ nanoparticle, Photocatalytic degradation, Sol–gel impregnation, Taguchi method

## Abstract

In this study the optimum conditions for preparing the iron-doped TiO_2_ nanoparticles were investigated. Samples were synthesized by sol–gel impregnation method. Three effective parameters were optimized using Taguchi method, consisted of: (i) atomic ratios of Fe to Ti; (ii) sintering temperature; (iii) sintering time. The characterization of samples was determined using X-ray diffraction, BET- specific surface area, UV- Vis reflectance spectra (DRS) and scanning electron microscope (SEM). The XRD patterns of the samples indicated the existence of anatase crystal phase in structure. UV- Vis reflectance spectra showed an enhancement in light absorbance in the visible region (wavelength > 400 nm) for iron-doped samples. The photocatalytic activity of samples was investigated by the degradation of RO 16 (RO 16) dye under UV irradiation. The results illustrated that the photocatalytic activity of iron-doped TiO_2_ was more than pure TiO_2_, because of the smaller crystal size, grater BET surface area and higher light absorption ability.

## Introduction

**Figure 1:**
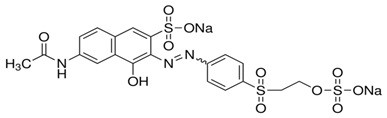
RO 16chemical structure.

Nowadays, increasing consumption of the wide variety of synthetic dyes in fabric dyeing, paper printing, color photography and product industrial contamination from the effluents has become significantly warning day by day. Azo-dyes build a high portion of synthetic dyes. Photodegradation of various azo-dyes have been reported like RO 16 (RO 16) (Figure 1). They are more poisonous and resistant to destroy by biological treatment methods [1-4]. Recently, the heterogeneous photocatalytic process has been a rapidly growing research area for the purification and complete mineralization of organic pollutants in industrial waste water and air [5].

The unique properties of titanium dioxide (TiO_2_) such as high photocatalytic activity, high chemical stability and low toxicity have made it a suitable photocatalyst in recent decades [6]. However, it is unavoidable to face two issues to make it the favorite catalyst for this process. One of which is to improve the low photo-quantum efficiency of TiO2 that arises from the fast recombination of photo-generated electrons and holes. The other, is to extend its photocatalytic activity into the visible light region in order to use solar energy as the light source [7]. Some researchers studied the photocatalytic activity of TiO2 in the visible light region. It can only make use 3–5% of the solar spectrum that reach earth because of its large band gap (Ebg, anatase ≅ 3.2 ev, rutile ≅3.0 ev ) [8], so it is essential to produce visible light responsive (VLR) TiO2, not only to use visible light but also to reduce the recombination of photo-generated electrons and holes [7]. In recent years some of the studies reported the possibility of producing these characteristics by chemical additives such as noble metals and ion doping. To reach these ends the use of noble metals such as gold [9], silver [10-13], transition metals such as Fe [5-9], Cr [14], Cu [15], Mn [16], Zn [17,18], V [19-22], W [23-25], non-metals such as nitrogen [26-29], carbon [29], has been reported. TiO_2_ particles can be doped by Iron to form a mixture of solids in low Iron concentration. Considering the similar radius of Fe^3+^ and Ti^4+^ ions (respectively 0.64 Å and 0.68 Å), titanium position in the lattice of TiO_2_ can be replaced by Iron cations easily. It has been found that the catalytic activity of these mixed oxides strongly depends on the preparation method, sintering temperature, sintering time and dopand content [5].

The aim of the present study is to synthesize conditions of Fe/Ti mixed oxides such as sintering temperature, sintering time and atomic ratios of Fe to Ti have seldom been optimized by Taguchi method, which is our motivation behind this study. Then, characteristics of them have been analyzed by XRD, DRS, SEM and BET. Next, catalysts which are synthesized have been investigated by degradation of Reactive Orange 16.

## Experimental

### Materials

The raw materials used in this study were HCl (30% w/w ), iron (III) acetylacetonate for synthesis and isopropanol for analysis, which were purchased from Merk, and titanium (IV) isopropoxide, which was purchased from Aldrich (99.9%). The azo dye, RO 16, was obtained from Ranginkaman Company. The molecular structure of RO 16 is shown in Figure 1.

### Experimental procedure

Nano sized binary mixed oxide of Fe/Ti was prepared by the sol–gel impregnation method [5]. There are three controllable parameters have been used for the synthetic procedure which each parameter has three levels (Table 1). Also, the experimental conditions are presented in Table 2[30,31]. The synthetic procedure follows a typical sol–gel impregnation method to prepare 1/1 molar composition of Fe/Ti mixed oxide. Under constant magnetic stirring 2.3 mL of titanium tetraisopropoxide was added drope-wise to a beaker containing 20 mL of iso-propanol and 1 mL HCl, while the beaker was maintained at 0°C. In another beaker similarly 2.77 mg Fe (III) acetylacetonate was dissolved in 20 mL iso-propanol and then was mixed drop-wise to the first beaker with vigorous stirring. The mixture was stirring in the 25°C for 24 h. The mixed sol was then sonicated in an ultrasonic (up 200 s, Hielscher, Germany) at room temperature for 15 min. The sonicated sol was placed over a hotplate to remove solvent. The resulting powder was calcinated in specified temperature and time. Nano sized titanium dioxide was prepared in a similar approach. Under constant magnetic stirring 2.3 mL of titanium tetraisopropoxide was added drop-wise to a beaker containing 20 mL of a solvent such ISO-propane and 1 mL HCl, whereas the temperature of beaker was kept at 0°C with a water and ice bath. The mixture was stirred at 25°C for 24 h. The resulting sol was then sonicated for 15 min and then its solvent was removed over a hotplate with a rather slow rate. The powder was calculated at the desired temperature and time. The calcinated powders were then grounded in mortar pestle and were characterized by XRD, DRS, SEM and BET surface area. Eventually, the photocatalytic activity of the samples was determined by the degradation of RO16 azo dye.

**Table 1:** Controllable parameters and their levels

**Factor**	**Description**	**Level 3**	**Level 1**	**Level 2**
A	atomic ratios of Fe to Ti (%)	0.2	0.1	0.05
B	Temperature of calcination (°C)	550	450	500
C	Time of calcination (h)	10	5	7.5

**Table 2:** Test conditions

**Test**	**Atomic ratios of Fe to Ti (%)**	**Temperature of calcination (°C)**	**Time of calcination (h)**
Test 1	0.1	450	5
Test 2	0.1	500	7.5
Test 3	0.1	550	10
Test 4	0.05	450	7.5
Test 5	0.05	500	10
Test 6	0.05	550	5
Test 7	0.2	450	10
Test 8	0.2	500	5
Test 9	0.2	550	7.5

### Photoreactor and light source

**Figure 2:**
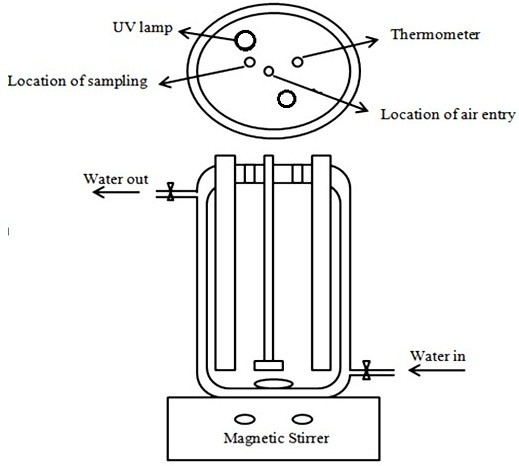
Schematic representation of photoreactor.

The photocatalytic activity of Test1-Test9 samples was determined by degradation of RO16. Experiments were carried out in a flask batch cylindrical photoreactor. The ports for sampling and oxygen purging were improvised on the photoreactor lid. In order to prevent radiation release, the outside wall of photoreactor was covered with aluminum foil. Two UV light lamps (6 W, OEM, China) were placed at the center of the reactor as a radiation source. In order to control the temperature of the reactions, the reactor was provided with a jacket for water circulation the reactor used in this study is represented in Figure 2. For each of the Test1-Test9 samples 100 cc (80 ppm) of aqueous solution of RO16 was mixed with 100 mg of the samples. The solution was transferred to the photoreactor, aerated and stirred in the darkness to reach suitable adsorption of RO16 on the surface of photocatalyst particles. After 30 min the lamps were turned on and the degradation reaction was initiated. Stirring and air sparging were kept on during the reaction. In order to analyze the concentration of dye, 3 cc of the solution inside the reactor were taken out after 60 min and centrifuged for catalyst separation. The catalyst loading (1 g/L), light intensity (12 W) and solution pH (2) were constant for all Test1-Test9 tests.

The concentration of RO16 in the samples was determined using UV/Vis spectrophotometer (CECIL2501) at 494 nm wavelength.

## Results and discussion

### X-ray diffraction analysis

**Figure 3:**
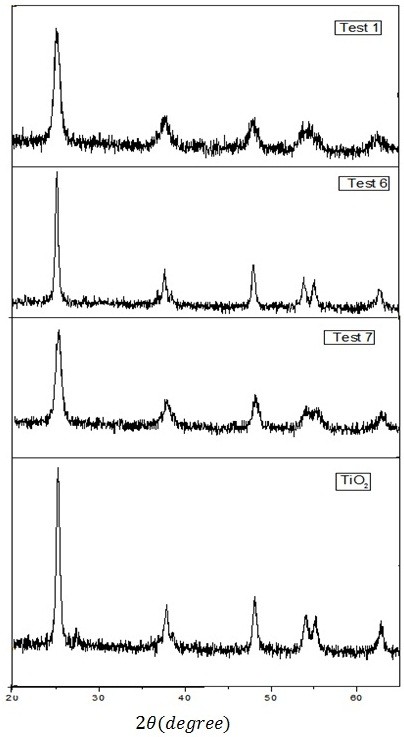
XRD patterns of some test1-test9 samples.

The composition of the crystalline phase and the average size of the crystals in the samples were determined by XRD. Figure 3 shows XRD patterns of some Test1-Test9 samples. The XRD spectrum of the samples shows a peak at 2*θ* ≅ 25.3 which represents the anatase crystalline phase. Iron can easily occupy the titanium position in TiO_2_ lattice, because of the similar ionic radius of Fe^3+^ and Ti^4+^, thereby there is no crystalline phase containing Iron in XRD patterns. Another reason can be the low concentration of Iron in the composition of binary Fe/Ti mixed oxide. These results confirm that this doping method has produced a uniform distribution of the Iron and has formed a homogeneous solid solution with TiO_2_. The XRD patterns indicate that the crystal size of the samples has been in the nano scale because the peaks are very broad. Also it can be calculated from the Debye-Scherrer formula [7]:

(1) $$D=\frac{K\lambda }{\beta \;\cos\theta }$$

Where D is average crystal size, K is a constant between 0.8-1 and is taken 0.9 for present calculations, λ is wavelength of XRD (1.54 Å), θ is the diffraction angle and β is full width at half maximum (FWHM). The calculated crystals size (Table 3) specify that doping iron with suitable concentration can decrease the crystal size. When Fe^3+^ ions are penetrated in the TiO_2_ lattice, some extent deformation will be occurred in the crystal lattice of TiO_2._ This lattice deformation and different ionic radius of Fe^3+^ and Ti^4+^ may result in decrease of crystals growth.

**Table 3:** **Particle characteristics of the undoped and doped TiO**_**2**_

**Sample**	**Crystal phase**	**Crystallite size (nm)**	**Surface area**$\left(\frac{m^{2}}{\mathrm{gr}}\right)$
Test 1	Anatase	11.46	76.34
Test 2	-	-	60.34
Test 3	-	-	42.13
Test 4	-	-	66.61
Test 5	-	-	48.39
Test 6	Anatase	22.92	39.59
Test 7	Anatase	10.77	80.47
Test 8	-	-	55.69
Test 9	-	-	42.93
TiO_2_	Anatase	18.85	41.2

### SEM and BET analysis

**Figure 4:**
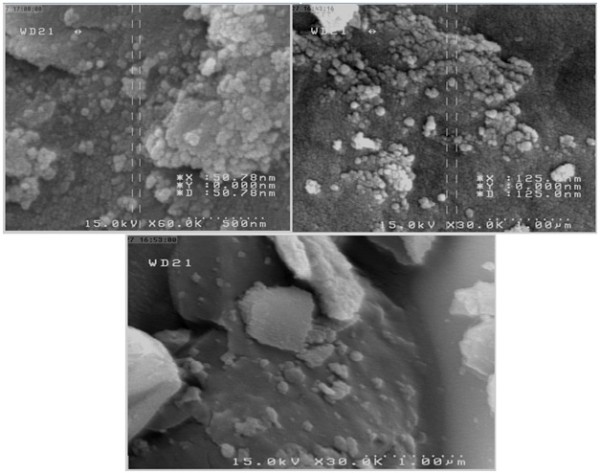
SEM images of some samples.

Figure 4 shows the SEM images of some Test1-Test9 samples. It is evident that the surface morphologies for these samples are approximately similar and spherical shape with non-uniform sizes. The size of particles varies from 50 to 200 NM which shows that a particle is formed from many crystals. Also it was seen that particle agglomerations are commonly formed due to this synthesis method.

The BET surface area for all Test1-Test9 samples was determined using 3-points method. These results are presented in Table 3. The BET surface area increases with increasing the iron content in the TiO_2_ lattice in a similar temperature. It is related to the decreased crystals and particle size due to distribution of iron dopant in TiO_2_.

### UV–Vis diffuse reflectance spectroscopy

**Figure 5:**
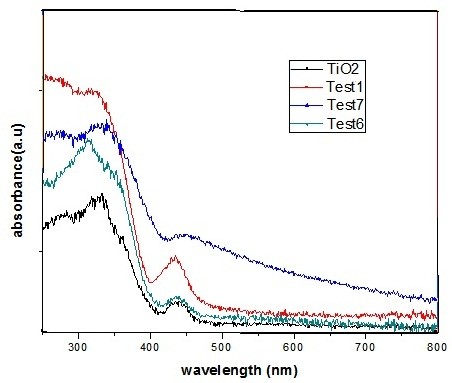
The UV–vis reflectance spectra of some samples.

The optical properties of some samples were determined by UV–vis diffuse reflectance spectra (DRS), and the results were presented in Figure 5. An increase of the light absorption at 400 nm can be related to the inherent band gap absorption of anatase titanium dioxide (~3.2 ev). It has been found that the light absorption of undoped TiO_2_ in the visible region (>400 NM) was not significant, whereas iron-doped TiO_2_ shows an enhancement of light absorption in this region. The light absorption in visible region was increased with increasing the iron concentration due to the changes in color from white to light yellow.

### Optimum conditions for photocatalytic degradation of RO16

The degradation efficiency of each test was determined using the following correlation:

(2) $$Y=1-C/C_{0}$$

Where C_0_ and C are initial and final concentrations of RO16 respectively. The degradation efficiency of each test is listed in Table 4. Each test was repeated three times to increase the accuracy of the results. The degradation efficiency and the number of repetitions were substituted into Eq. (1), and $\left(\frac{S}{N}\right)$ ratio of each test was determined. To obtain the mean value of the $\left(\frac{S}{N}\right)$ ratios of a certain factor in the *ith* level, $M_{\text{level}}^{\text{factor}}$ (Table 5), the $\left(\frac{S}{N}\right)$ ratios of Table 4 were substituted in Eq. (2). Table 5 suggested that a condition of sintering temperature at 450°C, sintering time of 5 h and atomic ratios of Fe to Ti 0_/_1% could be a good condition to achieve the best dye removal.

**Table 4:** **The**$\left(\frac{S}{N}\right)$**ratio of each test**

**Test**	**Atomic ratios of Fe to Ti (%)**	**Temperature of calcination (°C)**	**Time of calcination (h)**	**Y**_**1**_**%**	**Y**_**2**_**%**	**Y**_**3**_**%**	$\left(\frac{S}{N}\right)$**average**
Test 1	0.1	450	5	93	92	94	39.3687
Test 2	0.1	500	7.5	92.0	94.0	93.0	38.9113
Test 3	0.1	550	10	87.2	89.4	88.1	37.9841
Test 4	0.05	450	7.5	80.4	79.0	78.5	38.7957
Test 5	0.05	500	10	88.2	86.0	87.0	38.0777
Test 6	0.05	550	5	81.5	79.0	80.0	37.8086
Test 7	0.2	450	10	79.0	76.0	78.2	39.0261
Test 8	0.2	500	5	88.5	90.0	89.7	38.1667
Test 9	0.2	550	7.5	82.5	79.5	81.0	37.0562
TiO_2_	-	450	4	72.4	70.8	69.87	-

**Table 5:** S/N ratio response table

$\frac{\text{factor}}{\text{level}}$	${\left[{\left(\frac{S}{N}\right)}_{\mathrm{factor}}^{\mathrm{level}}\right]}_{j}$			$M_{\mathrm{factor}}^{\mathrm{level}}$
	**j = 1**	**j = 2**	**j = 3**	
**A/1**	39.3687	38.9113	37.9841	38.7547
**A/2**	38.7957	38.0777	37.8086	38.22
**A/3**	39.0261	38.1667	37.0562	38.083
**B/1**	39.3687	38.7957	39.0261	39.0635
**B/2**	38.9113	38.0777	38.1667	38.385
**B/3**	37.9841	37.8086	37.0562	37.6163
**C/1**	39.3687	37.8086	38.1667	38.448
**C/2**	38.9113	38.7957	37.0562	38.2544
**C/3**	37.9841	38.0777	39.0261	38.3626

Y_1_, Y_2_ and Y_3_ represent the experimental results (degradation of pollutant) at first, second and third test pieces respectively. The boldface corresponds to the maximum of $\left(\frac{S}{N}\right)$ ratio among the 9 tests. It is observed that the photocatalytic degradation of RO 16by Fe-TiO_2_ which is synthesized in the best condition, which is mentioned above, significantly (nearly 93 percent) more than that of TiO_2_ (approximately 71 percent) (see Table 4). This results from characteristics of catalyst Fe-TiO2 which include smaller size of catalyst, bigger surface area of catalyst, higher absorption of light by catalyst in comparison with TiO2.

## Conclusion

Binary mixed oxide of Fe/Ti was prepared using the sol–gel impregnation method. The characterization showed that the mixed oxide always consists of amateurs phases of TiO_2_ and there are no rutile phase of TiO_2_ and iron phase. SEM images revealed the similar morphology of samples with 50–300 nm particle size and the BET analysis indicated that the samples with more iron dopant in a similar calcination temperature have greater surface area. The optimum conditions were 450°C for sintering temperature, 5 h for sintering time and 0_/_1% for atomic ratios of Fe to Ti. The photocatalytic activity of iron-doped TiO_2_ has been improved compared to pure TiO_2_ because of the smaller crystallite size, greater BET surface area and higher light absorption ability.

## Competing interests

The authors declare that they have no competing interests.

## Authors’ contributions

MS and RT designed and performed experiments, analyzed data and wrote the paper; MHR performed the experiments, revised and edited the manuscript; MN and MD gave technical support and conceptual advice. All authors read and approved the final manuscript.
